# A Manual of Obstetric Practice

**Published:** 1898-09

**Authors:** 


**A Manual of Obstetric Practice.** By A. Dührssen, M.D. Translated from the Sixth Edition by John W. Taylor and Frederick Edge, M.D. Pp. xviii., 304. London: H. K. Lewis. 1897.

The author does not burden this work with useless detail; every practical point on which he lays stress is stated as an axiom and cannot fail to impress the mind of the reader. Some of the plates, however, do not appear as clear as they might, notably those attached to the description of the suture of the episiotomy incision; it is questionable whether anyone without a practical acquaintance with this subject could gain any assistance from figures of so sketchy a nature. The treatment of eclampsia is a subject which the author has made peculiarly his own: his practice is to deliver immediately, or as soon as possible if eclampsia has already set in, and to do this under deep anæsthesia. In cases of *post-partum* hemorrhage with atony the author recommends the plugging of the uterus with iodoform gauze preferably, or other material if this is not at hand: this plan of treatment no doubt would succeed if other measures failed, but it should be impressed on the reader that it is not the simple proceeding it is often thought to be.

